# The Relationship Between Preparedness for Caregiving and Spiritual Well-Being in the Carers of Stroke Patients: A Case Study in Türkiye

**DOI:** 10.1007/s10943-024-02033-4

**Published:** 2024-04-16

**Authors:** Fatma Özkan Tuncay

**Affiliations:** Department of Medical Nursing, Health Sciences Faculty, Cumhuriyet University, 58140 Sivas, Turkey

**Keywords:** Stroke, Caregiving, Preparedness, Spiritual well-being

## Abstract

This study was conducted to determine the relationship between preparedness for caregiving and spiritual well-being in the carers of stroke patients. This descriptive and cross-sectional study was conducted with 130 primary carers of patients hospitalized with the diagnosis of stroke at the neurology clinic of a hospital. It was determined that the participants had moderate levels of preparedness for caregiving, they had high levels of spiritual well-being, and there was a positive significant relationship between these two variables. According to the regression analysis results, the spiritual well-being level of the participants was a predictor of their preparedness for caregiving (*B*: 0.144, *p* < 0.001). The results of this study demonstrated that the spiritual well-being of the carers of stroke patients is important in increasing preparedness for caregiving. In this context, to ensure that the carers of all stroke patients feel prepared for the caregiving process, it is recommended to evaluate their preparedness levels, support them in their preparation for their caregiving roles, and identify their spiritual needs.

## Introduction

Stroke is a health problem that is accepted to be the most frequently encountered cause of morbidity and mortality worldwide and causes multiple physical and social limitations (Anoopa & Vijayalakshmi, 2017). The incidence of stroke is estimated to be between 7.5 and 10.1 in every 1000 people (Gabet et al., 2021). Stroke causes different changes in the lives of both the patient and their family throughout its course. Approximately 80% of stroke patients are dependent on their families in their daily activities, and thus, the carers of patients who experience a stroke frequently face the burden of providing care for these patients (Pucciarelli et al., 2022).

In addition to its positive aspects such as personal growth, satisfaction, receiving social support from others, and self-esteem, caregiving also leads to the experience of several difficulties. These problems may lead to increased rates of errors in care, ill-advised and inappropriate interventions, delays in recovery, and repeated hospitalizations.

The preparedness level of a person is associated with whether their experiences regarding caregiving are perceived as positive or negative (Henriksson et al., 2015; Pucciarelli et al., 2014). Preparedness is a concept that is associated with how individuals perceive the difficulties in their caregiving roles such as providing physical care, providing emotional care, and coping with caregiving stress. Preparedness is also defined as a concept that can protect the carer from experiencing a caregiving burden and reduce negative outcomes associated with caregiving (Chafjiri et al., 2017; Shyu et al., 2010).

It was stated that carers who perceived themselves to be unprepared for caregiving perceived higher levels of carer burden, their daily activities such as sleep and rest were affected, and they faced various issues such as chronic fatigue, financial problems, and depression (Chafjiri et al., 2017; Gholamzadeh et al., 2014; Henriksson et al., 2015).

On the other hand, knowing how to help stroke patients who have various limitations and being prepared in this process makes the adaptation of carers to changes emerging in their own lives easier. In the literature, it was reported that the carers of patients with neurological disorders experienced high levels of emotional stress (Pucciarelli et al., 2014; Shaffer et al., 2016). For this reason, to protect the patient and their cares from the negative effects of the caregiving process, it is important for healthcare professionals to assess the preparedness statuses of carers in this new role they are encountering suddenly and without a warning, as well as the variables affecting this issue (Pucciarelli et al., 2022).

One of the important factors that affect adjustment to and preparedness for caregiving among carers is the spiritual tendencies of these individuals (Chafjiri et al., 2017). Spirituality is an important resource to protect mental health and is considered a mechanism for coping with stressful life events (Ekşi & Kardaş, 2017; Martínez & Custódio, 2014). Spiritual well-being is defined as the feeling of communicating with others, finding meaning in life, believing in a greater power, and interacting with that power (Uğurluoğlu & Erdem, 2019). The concept of spiritual well-being may help individuals hold on to life, love themselves and nature, stand more resilient against traumatic events by believing in the existence of a superior power, and be more involved in life (Erişen & Sivrikaya, 2017; Uğurluoğlu & Erdem, 2019).

Studies in the literature have reported that spiritual well-being has favorable effects on illness and health (Bonelli & Koening, 2013; Bravin et al., 2019). It was stated that individuals, with high levels of spiritual well-being have a healthy lifestyle, are more satisfied with their lives, and cope with the difficulties they encounter more easily (Ekşi et al., 2019; Mahdian & Ghaffari, 2016). It was emphasized that spirituality gives people the strength to understand the negative events they experience and overcome the traumas created by these events, gives meaning to their suffering, provides them with hope, and contributes to the psychological resilience of individuals (Ekşi et al., 2019).

Researchers focusing on spiritual needs have found that in general, spirituality helped the carers of individuals with chronic diseases cope with their daily lives and feelings of being underwhelmed by the physical and emotional aspects of care (Duran et al., 2020; Eslami et al., 2014; Pucciarelli et al., 2022). Furthermore, spiritual anxieties or unmet spiritual needs may lead to a variety of feelings including distress and unnecessary physical and emotional challenges, and they may prevent people from feeling prepared for caregiving responsibilities. It was found that high intrinsic spirituality in carers enabled them to devote more time to caregiving and protected them against the emotional distress related to the provision of help. It has been stated that with spiritual well-being, the willingness level of the carer to provide care increases, and the carer accepts this responsibility and prepares themselves more for this role (Dikmen & Üşenmez, 2022; Lai et al., 2018; Lalani et al., 2019).

Stroke is a chronic condition that necessitates multidimensional and overwhelming care. The carers of stroke patients face various stressors that can threaten different aspects of their health, especially their mental health. Spiritual attitudes and being spiritually oriented contribute significantly to mental health and can be used as a strategy for adapting to stressful events brought about by caregiving roles (Anoopa & Vijayalakshmi, 2017; Chafjiri et al., 2017). Additionally, caregiving preparedness is very important for carers after a case of stroke to learn how to help individuals with multiple deficits quickly and adapt to the changes that occur in their lives as a result of caregiving (Gholamzadeh et al., 2014).

In this context, the preparedness and spiritual well-being of individuals may affect the management of patient care in a better or worse direction (Erişen & Sivrikaya, 2017). Despite their positive effects on issues that are encountered during the caregiving process, both concepts are neglected in not only clinical practice but also scientific research. Furthermore, it is seen that there is little research on the relationship between caregiving preparedness and spiritual well-being in carers in general, whereas there is no study on this topic in Türkiye. In this sense, the purpose of this study is to determine the caregiving preparedness and spiritual well-being levels of the carers of stroke patients and investigate the relationship between these two variables.

It is believed that the results obtained in this study will fill a gap in the relevant literature and guide nurses in increasing the caregiving preparedness and spiritual well-being levels of the carers of stroke patients. Additionally, it is thought that these results will be meaningful for increasing the awareness of healthcare professionals about preparedness, as well as spirituality affecting the preparedness, which is a very important issue in the caregiving process but has not been included much in the clinical field, and consequently, for supporting carers.

## Methods

### Design

This is a descriptive and cross-sectional study.

### Sample

The sample of this study included 130 primary carers of patients diagnosed with stroke and hospitalized in the Neurology Clinic of a University Hospital between November 2022 and April 2023. A primary carer was defined as the person who lives with the patient in the same house and takes the primary responsibility for providing care to the patient at home (Şirzai et al., 2015). The sample size required to conduct the study was calculated with the PASS (Power Analysis and Sample Size) 11 Statistical Analysis Software (NCSS LLC, Kaysville, Utah, USA). The minimum sample size was calculated as n = 127 by using alpha (a) = 0.05, beta (b) = 0.20, and 1-b = 0.80. The carers of patients who were diagnosed with stroke for the first time and who were admitted to the neurology clinic between the aforementioned dates were included in the sample.

To participate in the study, carers were required to be 18 years of age or older, be literate, have no cognitive impairments, and agree to participate in the study after they were informed about the study. The exclusion criteria were being the carer of a patient who had been diagnosed with stroke before, being the carer of a patient with a diagnosis of a secondary physical or mental illness, having perception disorders, and having communication problems.

### Data Collection Tools

Data were collected using a Personal Information Form, the Preparedness for Caregiving Scale, and the Spiritual Well-Being Scale. The independence level of the patient group in their activities of daily living was determined using the modified Barthel Index.

#### Personal Information Form

The form that was prepared by the researchers contained a total of 16 questions including 10 questions on the sociodemographic information of the participants such as their age, gender, marital status, education status, and duration of caregiving, and 6 questions regarding information about the patients who were being provided care by the participants.

#### Preparedness for Caregiving Scale (PCS)

The scale was developed by Archbold et al. in 1990 and is an 8-item self-assessment standard scale for carers. In this scale, preparedness is defined as a perceived readiness for the various aspects of caregiving, e.g., providing physical care, psychological support, and coping with the stress and pressure of home-based caregiving. Each item has 5-point Likert-type response options, ranging from 0 to 4 (0 = not at all prepared to 4 = very well prepared) (Archbold et al., 1990).

The minimum and maximum scores that can be obtained from PCS are 0 and 32. Higher PCS scores indicate that the respondent feels more prepared to provide care. The validity and reliability study of the scale in Türkiye was conducted by Karaman and Karadakovan (2015). In the study, the Cronbach’s alpha internal consistency coefficient of the scale was found to be 0.84.

#### Spiritual Well-Being Scale (SWBS)

The scale was developed by Eksi and Kardas (2017) to evaluate people’s processes of understanding and living their lives with their personal, social, environmental, and transcendental aspects. In the Turkish validity and reliability study of the scale, Cronbach’s alpha coefficient was determined as 0.88. The 5-point Likert-type scale consists of 29 items and three dimensions as transcendence, harmony with nature, and anomie (Appendix 1). The score range of the scale is 29–145, and higher scores indicate higher levels of spiritual well-being (Eksi & Kardas 2017). The Cronbach’s alpha coefficient of the scale was found as 0.82 in this study.

#### Modified Barthel Index (MBI)

The index was developed by Mahoney and Barthel in 1965. The validity and reliability study of the index for the Turkish population was carried out with neurology patients by Küçükdeveci et al. (2000). The Barthel Index is used to determine an individual’s level of independence in performing activities. The performance of patients is determined by observation. Index scores can range from 0 to 100, where 0 indicates complete dependence, and 100 indicates complete independence.

### Data Collection

Carers were given information about the purpose and scope of the study, and those who met the inclusion criteria provided written consent before their inclusion. The data were collected by the researchers in face-to-face interviews with the participants in a suitable meeting room at the clinic, and each interview lasted about 20–25 min.

### Ethics Approval

The study was performed in line with the principles of the Declaration of Helsinki and approved by the Cumhuriyet University Clinical Research Ethics Board (decision no: 2022–10/15). After the participants were informed about the study and ensured that the data to be collected would be kept confidential, their verbal and written consent was obtained.

### Data Analysis

For data analysis, the SPSS 22.0 software (SPSS, Inc., Chicago, IL, USA) was used. Normality was analyzed with the Kolmogorov–Smirnov test. Since the data were normally distributed, t-tests were used for pairwise comparisons, and one-way analysis of variance (ANOVA) was used for multiple comparisons (Tukey’s b test for post hoc comparisons). Pearson’s correlation analysis was used to investigate the relationships between the preparedness and spiritual well-being levels of the participants. Multivariate linear regression analysis was used for the variables predicting the preparedness levels of the participants. For the significance level of the statistical tests, *p* < 0.05 was determined.

## Results

### Participant Characteristics

It was determined that 70.0% of the participants were women, 34.6% were at the ages of 45–64 years (mean age: 47.95 ± 12.41 years), 65.4% had primary-secondary school degrees, 53.8% were providing care for their children, and 54.6% had been carers for the last 0–6 months. Considering the patient group provided with care, it was found that approximately half of the patients (45.4%) were severely dependent (Table 1).

**Table 1:** Demographic characteristics of participants

	Descriptive characteristics	
	*n*	%
Gender		
Women	91	70.0
Men	39	30.0
Age (*X* ± SD: 47.95 ± 12.41)		
22–44 years	41	31.5
45–64 years	45	34.6
≥ 65 years	44	33.9
Educational status		
Illiterate or literate with no formal degree	13	10.0
Primary-secondary school	85	65.4
High school or higher	32	24.6
Relationship with patient		
Parent	17	13.1
Spouse	43	33.1
Son/daughter	70	53.8
Caregiving duration		
0–6 months	71	54.6
7–13 months	24	18.5
≥ 14 months	35	26.9
MBI (patient) *X* ± SD: 53.30 ± 27.20		
Complete dependence	21	16.2
Severe dependence	59	45.4
Moderate dependence	32	24.6
Mild dependence	18	13.8

### Preparedness for Caregiving and Spiritual Well‑Being

The mean PCS score of the participants was 21.22 ± 6.33. The total Spiritual Well-Being Scale (SWBS) scores of the participants varied between 29 and 145, while their mean score was 114.63 ± 13.14. The mean transcendence, harmony with nature, and anomie subscale scores of the participants were 63.65 ± 8.04, 27.86 ± 4.16, and 23.11 ± 4.70, respectively (Table 2).

**Table 2:** Mean PCS and SWBS scores of the participants

Scales	Mean ± SD	Min–Max	Score range
Preparedness for Caregiving Scale	21.22 ± 6.33	8–32	0–32
Spiritual Well-Being Scale	114.63 ± 13.14	80–145	29–145
Transcendence subscale	63.65 ± 8.04	45–75	15–75
Harmony with nature subscale	27.86 ± 4.16	20–35	7–35
Anomie subscale	23.11 ± 4.70	9–34	7–35

### Caregiving Preparedness and Spiritual Well-Being Levels Compared Based on Some Characteristics

Table 3 shows the distributions of the caregiving preparedness and spiritual well-being levels of the participants based on some variables. While the caregiving preparedness levels of the female participants and those with degrees from vocational schools of higher education were significantly higher, the mean PCS score of those who had caregiving durations of 0–6 months was significantly lower (*p* < 0.05). In the comparisons of the scores of the participants based on the dependency levels of their patients, it was seen that the mean SWBS score of the participants providing care for patients with low levels of dependency was significantly lower, while their mean PCS score was significantly higher (*p* < 0.05).

**Table 3:** Comparisons of PCS and SWBS scores based on some characteristics of the participants

Characteristics	SWBS Mean ± SD	PCS Mean ± SD
Gender		
Women	115.86 ± 12.15	23.30 ± 6.66
Men	117.76 ± 14.97	20.32 ± 5.99
Test (t/p)	1.640/0.103	2.508/**0.013**
Age (years)		
22–44	112.21 ± 11.75	20.97 ± 5.54
45–64	116.71 ± 12.44	21.91 ± 6.37
≥ 65	114.77 ± 14.86	20.75 ± 7.02
Test (F/p)	1.262/0.287	0.416/0.661
Education level		
Illiterate or literate with no formal degree	113.07 ± 6.55	21.69 ± 8.50
Primary-secondary school	114.14 ± 13.28	20.38 ± 6.04
High school or higher	116.59 ± 14.75	23.25 ± 5.78
Test (F/p)	0.503/0.606	2.740/**0.047**
Relationship with patient		
Parent	115.52 ± 11.79	21.58 ± 6.76
Spouse	117.30 ± 15.38	21.74 ± 5.85
Son/daughter	112.78 ± 11.76	20.81 ± 6.56
Test (F/p)	1.634/0.199	0.316/0.729
Caregiving duration		
0–6 months	116.49 ± 11.78	18.00 ± 7.13
7–13 months	114.66 ± 8.17	21.66 ± 5.73
≥ 14 months	110.85 ± 17.38	22.54 ± 6.35
Test (F/p)	2.196/0.115	4.245/**0.016**
MBI (patient)		
Complete dependence	117.20 ± 11.68	19.66 ± 6.03
Severe dependence	115.95 ± 12.26	22.52 ± 7.89
Moderate dependence	114.22 ± 11.24	21.31 ± 6.63
Mild dependence	109.28 ± 15.91	24.66 ± 1.37
Test (F/p)	2.701/**0.048**	3.457/**0.019**

Bold values are statistically significant (*p* < 0.05)

PCS, Preparedness for Caregiving Scale; SWBS, Spiritual Well-Being Scale; t, Independent sample t-test; F, One-way analysis of variance

### Relationship Between the Caregiving Preparedness and Spiritual Well-Being Levels of the Participants

The correlations between the mean PCS scores of the participants and their mean total SWBS and SWBS subscale scores are presented in Table 4. The Pearson’s correlation analysis revealed a weak, positive, and statistically significant relationship between the PCS scores of the participants and their SWBS total and subscale scores (SWBS total r: 0.372, Transcendence r: 0.383, Harmony with nature r: 0.381, Anomie r: 0.315; *p* < 0.01).

**Table 4:** Correlations between preparedness for caregiving and spiritual well-being

Variables	SWBS total	Transcendence	Harmony with nature	Anomie
PCS				
*r*	0.372	0.383	0.381	0.315
*P*	0.002	0.001	0.001	0.002

PCS, Preparedness for Caregiving Scale; SWBS, Spiritual Well-Being Scale

### Predictive Factors of the Caregiving Preparedness Levels of the Participants

The results of the multiple regression analysis, which was conducted to explain the factors that affected the caregiving preparedness levels of the participants, are shown in Table 5. According to the results, the spiritual well-being levels of the participants were a predictor of their preparedness for caregiving (*B*: 0.144, *p* < 0.001), but the dependency levels of their patients were not a significant predictor (*B*: 0.033). The spiritual well-being levels of the participants explained 7.9% of the total variance in their levels of preparedness for caregiving (adjusted *R*^2^: 0.79), meaning that these levels had a weak significant effect. The variables included in the analysis were gender, age, education level, caregiving duration, and the patient’s independence level (Table 5).

**Table 5:** Predictive factors of caregiving preparedness

Variables	B (95% CI)	SE	*β*	*t*	*p*
Constant	2.942	5.094	–	0.578	0.565
Spiritual Well-Being Scale	0.144	0.041	0.299	3.474	0.001
MBI (patient)	0.033	0.020	0.142	1.655	0.100

*R* = 0.306, Adjusted *R*^2^ = 0.79, *F* = 6.576, *p* = < 0.001

Adj. *R*^2^, Adjusted *R*-squared; *B*, partial regression coefficient; *β*, standard partial regression coefficient; 95% CI, 95% confidence interval

## Discussion

It is important to investigate the factors that affect care among carers and use the results that are obtained in patient care and the continuation of the well-being of the carer (Duran et al., 2020). In this study, which aimed to determine the caregiving preparedness and spiritual well-being levels of the carers of stroke patients, the mean PCS score of the participants was found as 21.22 ± 6.33. This mean score indicated that the participants had moderate levels of preparedness for caregiving in general. In another study that was conducted with the carers of stroke patients in Türkiye, the mean PCS score of the participants was determined to be 19.47 ± 7.40 (Aydın, 2021). Mohammadi et al. (2019) reported a mean PCS score of 22.46 ± 4.00. These results were in parallel with the literature. As a different result, in their study conducted with carers of stroke patients, Liu et al. (2020) determined that based on their mean score, carers were not sufficiently prepared to provide care. These differences may have stemmed from cultural differences and changes in perceptions of care.

In the literature, the importance of the preparedness of the carer in the recovery of the stroke patient was emphasized, and it was determined that preparedness alleviated depression in the care process and improved quality of life (Petrizzo et al., 2023; Pucciarelli et al., 2022). Studies carried out with older adults and individuals with dementia revealed that carers feeling prepared for their caregiving roles experienced less worry and had lower rates of depression (Lee et al., 2019; Supiano et al., 2022). In this sense, it can be stated that for the continuation of the well-being of both the patient and their carer, it is important to ensure that the carer is prepared for this role and determine the factors that influence this matter.

In the examination of the characteristics influencing caregiving preparedness in this study, it was seen that the female participants had significantly higher levels of preparedness for caregiving. A similar result was obtained in a study conducted in China (Liu et al., 2020). Studies performed with carers have mostly determined that women do not only have higher levels of preparedness for caregiving but also experience situations such as caregiving burden and caregiving stress more frequently (Bahadır & Ata, 2017; Liu et al., 2020; Şirzai et al., 2015). The reason for this may be that women take on the responsibility of caregiving in addition to their existing roles as spouses, mothers, and homemakers in many societies.

It was found that participants who graduated from high school or higher had significantly higher levels of preparedness for caregiving. Because carers with increasing levels of education, who have easier access to health-related information, have a higher chance to improve their knowledge and skills and receive more comprehensive information and medical help, they are more prepared to become carers (Pucciarelli et al., 2014). In contrast with these results, Shyu et al. (2010) found that the education levels of carers were not a significant factor in determining their preparedness for caregiving. This difference may be explained by the fact that most of the carers who participated in the study conducted by Shyu et al. had high levels of education.

Carers with low education levels may have substantially more insufficient knowledge of the causes of stroke, as well as its treatment and care, and this may affect their preparedness for caregiving (Liu et al., 2020). The higher awareness levels of the participants with higher levels of education, their usage of effective problem-solving skills, their easier access to information/healthcare services, and their better management of patient care could have contributed to the increase in their preparedness for caregiving.

In this study, the mean SWBS score of the participants, considered to reflect their spiritual well-being levels, was determined to be 114.63 ± 13.14. According to this result, the participants had high levels of spiritual well-being in general. Considering the SWBS subscale scores of the participants, it was seen that their spiritual well-being was rather based on their respect for nature, harmony with nature, and belief in the existence of a superior power.

The high spiritual well-being levels of the carers who participated in this study, which was performed in Türkiye, a country with a predominantly Muslim population, were promising in terms of suggesting that they would be able to positively manage the difficulties and burden of caregiving. It was previously reported that spiritual well-being acted as a coping and adjustment strategy and increased physical and mental health (Duran et al., 2020; Tavassoli et al., 2019).

In Türkiye, a study that included the carers of patients confined to bed reported a mean SWBS score of 115.09 ± 18.89, while another study carried out with the carers of cancer patients reported a score of 119.77 ± 22.91 (Kaplan & Beydağ, 2023, Semerci et al., 2023). It may be noticed that the results of different studies on the topic are very close to each other. This similarity can be attributed to the similarities in the beliefs of individuals living in Türkiye, their value judgments, and their methods of coping with challenging situations.

There are several studies on the spiritual well-being of carers, and spiritual well-being is accepted as an important concept that influences the quality of life of individuals (Lafcı et al., 2020; Semerci et al., 2023; Ugalde et al., 2019). It was also suggested that spiritual well-being had a significant effect on coping with the caregiving process and adjusting to it (Semerci et al., 2023).

Previous studies have shown that the dependency levels of patients affect the caregiving process. In this study, the dependency status of the individual who was being provided with care was identified as a factor that affected the caregiving preparedness and spiritual well-being levels of the participants. It was observed that as the dependency levels of their patients increased, the caregiving preparedness levels of the participants decreased, and their spiritual well-being levels increased.

Similar studies have also revealed that increased dependency levels of patients affect the preparedness levels of their carers (Aydın, 2021; Chafjiri et al., 2017; Shaffer et al., 2016). In this respect, it is needed to assess the dependency level of the patient who is being provided care and support the preparedness of carers, especially those looking after patients who are dependent, for caregiving.

In this study, it was found that spiritual well-being level of the participants who were carers of stroke patients increased, their preparedness for caregiving level also increased. This suggested that spiritual well-being was effective in caregiving preparedness. While, to the best of our knowledge, there is no study in the relevant literature that has examined the relationship between spiritual well-being and preparedness for caregiving, spiritual orientation is accepted as an important strategy for increasing adjustment in carers and preparing them for caregiving. Furthermore, there are also other studies showing that spiritual well-being reduces caregiving stress, caregiving burden, anxiety, and depression, and it raises quality of life (Gök Metin & Helvacı, 2020; Kaplan & Beydağ, 2023; Semerci et al., 2023). In the literature, it has been argued that spirituality is a significant strength and coping mechanism in coping with caregiving burden, and it provides hope for the future (Eksi et al., 2019; Li et al., 2018; Martinez & Custódio, 2014; Semerci et al., 2023).

While spiritual well-being is a unique tool in increasing the resilience of individuals against caregiving burden and preparing them for the caregiving process, it is thought that the spiritual needs of patients and their carer are not sufficiently met. Hence, nurses should keep in mind that they have a key role in raising the mental well-being levels of patients and individuals who provide care for patients with their care-providing, education, guidance, and supportive duties (Chafjiri et al., 2017).

### Limitations of the Study

This study had some limitations. First, due to cultural and religious differences, the results are specific to Turkish Muslim carers of stroke patients. Second, the representative power of the study is limited as it was conducted with the carers of stroke patients at a single university hospital. Lastly, as this study focused on a group of people who provide care for patients who usually consist of hospitalized individuals, follow-up studies may be needed.

On the other hand, this study is the first study in Türkiye to examine the relationship between the caregiving preparedness and spiritual well-being levels of the carers of stroke patients. It is expected to pave the way for approaches aiming to support the adjustment process of carers to the caregiving process and strengthen the coping of carers with problems brought about by caregiving burden.

## Conclusion and Recommendations

In this study, which aimed to examine the caregiving preparedness and spiritual well-being levels of the carers of stroke patients, it was determined that the participants had moderate levels of preparedness for caregiving, they had high levels of spiritual well-being, and spiritual well-being level of the participants increased, preparedness for caregiving level also increased. The results of this study demonstrated the importance of the spiritual well-being levels of the carers of stroke patients in terms of increasing their preparedness for caregiving.

In this context, to ensure that the carers of all stroke patients feel prepared for the caregiving process, it may be recommended to evaluate their preparedness levels, support them in terms of their preparedness for their caregiving roles, and identify their spiritual needs. To increase the preparedness of carers, it is recommended to create environments that increase the interaction of patients, carers, and family members with each other, support the carers in terms of providing care, and organize educational sessions involving other members of their family regarding the course, complications, and care management process of the disease.

It is important to establish environments at clinics that will make the spiritual practices of patients and their carers easier and support these individuals in this sense. Furthermore, prospective studies with larger samples can be conducted to explain the causal relationships between factors that affect preparedness for caregiving better and generalize the results to society. Additionally, it is recommended for clinical researchers to conduct interventional studies evaluating the effects of improved spirituality levels on preparedness by making plans to strengthen the spirituality of carers.

## Appendix 1: Instrument for Data Collection: Spiritual Well-Being Scale

**Table:**

		For each of the following statements, tick the option that best indicates how much you agree or disagree with the statement, based on a description of your personal experience 1 = Doesn’t suit me at all 2 = Doesn’t suit me 3 = Suits me a little bit 4 = Suits me quite well 5 = Suits me perfectly				
		(1)	(2)	(3)	(4)	(5)
1	Being connected to a divine power gives me safety					
2	I think nature should be respected					
3	I feel a sense of dissatisfaction about life					
4	I feel God’s help when I experience a problem					
5	I believe that God knows all my thoughts and feelings, secret and obvious					
6	I believe that all living creatures deserve respect					
7	There’s a big emptiness in my life					
8	I witness the power of God in my daily life					
9	I believe that God loves and cares for me					
10	I treat all living creatures on earth kindly					
11	I don’t enjoy life					
12	I feel God’s presence in every moment of my life					
13	The feeling of having protection from a more powerful being comforts me					
14	I see myself as part of the nature					
15	I still haven’t found the purpose of my life					
16	I believe there is a good in everything I go through					
17	My faith guides me how to live my life					
18	The rights of all living creatures on earth are important to me					
19	I wouldn’t know where to start to solve my problems					
20	When I am alone, I think of God and creation (I think and reason)					
21	My beliefs and values increase my resilience in the face of challenges					
22	I live in harmony with the nature					
23	When I experience difficulties, I feel overwhelmed					
24	My faith helps me to see that even in difficulties there could be a positive side					
25	Nothing in life is without a reason					
26	I think life consists of events that make me unhappy					
27	Knowing that not everything is in my control is a source of consolation in the face of upsetting events					
28	I believe that every natural creature on earth is unique					
29	Believing that the life of this world is temporary cleanses me of my ambitions					
